# Comparative chromosome maps between the stone curlew and three ciconiiform species (the grey heron, little egret and crested ibis)

**DOI:** 10.1186/s12862-022-01979-x

**Published:** 2022-03-03

**Authors:** Jinhuan Wang, Weiting Su, Yi Hu, Shengbin Li, Patricia C. M. O’Brien, Malcolm A. Ferguson-Smith, Fengtang Yang, Wenhui Nie

**Affiliations:** 1 State Key Laboratory of Genetic Resources and Evolution, Kunming Institute of Zoology, Chinese Academy of Sciences, Kunming, 650223 Yunnan People’s Republic of China; 2 Key Laboratory of Forensic Sciences, Ministry of Health, Xi’an Jiaotong University, Xi’an, 710061 People’s Republic of China; 3 Cambridge Resource Centre for Comparative Genomics, Department of Veterinary Medicine, University of Cambridge, Cambridge, CB3 0ES UK; 4 School of Life Sciences and Medicine, Shandong University of Technology, Zibo, 255049 Shandong People’s Republic of China

**Keywords:** Chromosome painting, Stone curlew, Herons, Ibises, Chromosomal rearrangements, Microchromosome fusion

## Abstract

**Background:**

Previous cytogenetic studies show that the karyotypes of species in Ciconiiformes vary considerably, from 2n = 52 to 78. Their karyotypes include different numbers of small to minute bi-armed chromosomes that have evolved probably by fusions of two ancestral microchromosomes, besides macrochromosomes and dot-like microchromosomes. However, it is impossible to define the inter-species homologies of such small-sized bi-armed chromosomes based on chromosome morphology and banding characteristics. Although painting probes from the chicken (*Gallus gallus*, GGA) chromosomes 1–9 and Z have been widely used to investigate avian chromosome homologies, GGA microchromosome probes are rarely used in these studies because most GGA microchromosome probes generated by flow sorting often contain multiple GGA microchromosomes. In contrast, the stone curlew (*Burhinus oedicnemus*, BOE, Charadriiformes) has an atypical low diploid chromosome number (42) karyotype and only 4 pairs of dot-like microchromosomes; a set of chromosome-specific painting probes that cover all BOE chromosomes has been generated. To get a genome-wide view of evolutionary chromosomal rearrangements in different lineages of Ciconiiformes, we used BOE painting probes instead of GGA painting probes to analyze the karyotypes of three ciconiiform species belonging to two different families: the eastern grey heron (*Ardea cinerea*, ACI, 2n = 64, Ardeidae), the little egret (*Egretta garzetta*, EGA, 2n = 64, Ardeidae) and the crested ibis (*Nipponia nippon*, NNI, 2n = 68, Threskiornithidae).

**Results:**

BOE painting probes display the same hybridization pattern on chromosomes of ACI and EGA, while a different hybridization pattern is observed on chromosomes of NNI. BOE autosome probes detected 21 conserved homologous segments and 5 fusions on the sixteen pairs of recognizable chromosomes of ACI and EGA, while 16 conserved homologous segments and 4 fusions were found on the twelve pairs of recognizable chromosomes of NNI. Only a portion of smaller bi-armed chromosomes in the karyotypes of the ciconiiform species could have evolved from fusions of ancestral microchromosomes. In particular BOE 5, which is the result of a fusion between two segments homologous to *GGA 7 and 8 respectively, was retained also as either a single chromosome in ACI (ACI 5) and EGA (EGA 5) or had fused with a part of the BOE 10 equivalent in NNI (NNI 5).*

**Conclusion:**

Our painting results indicate that different chromosome rearrangements occur in different ciconiiform lineages. Some of the small-sized bi-armed chromosomes in ACI, EGA and NNI are derived from the fusions of two microchromosomes, indicating that microchromosome fusions play an important role in ciconiiform chromosome evolution. The fusion segment homologous to GGA 7 and 8 is a potential cytogenetic signature that unites Ardeidae and Threskiornithidae.

## Background

According to traditional classification, the order Ciconiiformes comprises medium- to large-sized wading birds that have a worldwide distribution in temperate, subtropical, and tropical regions. There are 108 species in Ciconiiformes, belonging to three suborders, 5 families and 36 genera [1]. The five families include Ardeidae (herons, bitterns, and egrets), Balaenicipitidae (shoebills), Ciconiidae (storks), Scopidae (hammerkops), and Threskiornithidae (ibises and spoonbills). Up to the year of 2000, the karyotypes of more than twenty species in Ciconiiformes, have been reported consecutively [2–17]. Only the monotypic family Scopidae (hammerkops) has not been studied. The diploid numbers of ciconiiform species studied so far vary from 52 to 78. As found in most avian, the karyotypes of ciconiiform species are also composed of two groups of chromosomes that differ notably in size: macro- and microchromosomes (reviewed in [18, 19]). After comparing the morphology of the first three pairs of macrochromosomes in ciconiiform karyotypes, it was proposed that centric fission, pericentric inversion and centric fusion play a role in the karyotype evolution of Ciconiiformes [7, 16]. The first three macrochromosomes in Ciconiidae and Balaenicipitidae have retained their ancestral morphology, identical to that found in many other birds. The submetacentric third pair of macrochromosomes is the unique character for Ardeidae (which is subtelocentric in all other Ciconiiformes). The centric fission of the first pair of macrochromosomes was found only in Threskiornithidae. In addition, different numbers of medium to small-sized bi-armed chromosomes exist in species from different ciconiiform families [7]. However, the origin of these small-sized bi-armed chromosomes, and their inter-specific and inter-family homologies wait to be elucidated.

Cross-species chromosome painting has been utilized to detect chromosomal homologies between different avian species for more than two decades. Up to 2019, 96 avian species from eighteen orders have been analyzed by chromosome painting mainly using probes from the chicken (*Gallus gallus*, GGA) macrochromosomes 1–9 and Z, and four sets of probes from other bird species (reviewed in [20]). These molecular cytogenetic data have provided support for the suggestion that most avian species have an apparently conserved karyotype, only few rearrangements have occurred during about 100 million years of avian evolution [18, 21]. Comparative cytogenetic analyses have defined these evolutionary rearrangements as chromosomal fusions, fissions and inversions. These provide new insights into avian karyotype evolution and phylogenetic relationships (reviewed in [19, 22]). However, considering the huge number of extant avian species, only about 1% avian species have been analyzed by chromosome painting. Even in some orders, just two or three species have molecular cytogenetic data (reviewed in [20, 22]). For instance, in Ciconiiformes, only two storks have been studied recently using probes from GGA and *Leucopternis albicollis* (LAL) macrochromosomes [23], while species from the other four ciconiiform families have not been studied by chromosome painting.

The eastern grey heron (*Ardea cinerea*, ACI), the little egret (*Egretta garzetta*, EGA) and the crested ibis (*Nipponia nippon*, NNI) are species from two different ciconiiform families: the former two belong to the Ardeidae, the last is a member of the Threskiornithidae. Previous comparative cytogenetic study indicated that both Ardeidae and Threskiornithidae constitute a clear-cut group based on changes in chromosome morphology; however, such chromosomal changes could not provide information on the relationships between these families due to their potential independent origins and the presence of undefined small chromosomes involved in the fusion with a segment of chromosome 1 [7]. In avian cross-species chromosome painting studies, the most widely used painting probes are derived from GGA macrochromosomes 1–9 and Z, which only cover part of the avian genome, making it impossible to define the rearrangements involving microchromosomes. GGA microchromosomes are numerous and similar in size, it is difficult to generate chromosome-specific probes of single GGA microchromsomes using flow sorting and microdissection. While some GGA microchromosome painting probes are available, they are scarcely used in avian chromosome painting studies due to each containing multiple GGA microchromosomes. To study avian microchromosome evolution, the painting probes that cover the entire genome, including microchromosomes, of a given avian species are required. Probes from the stone curlew (*Burhinus oedicnemus*, BOE, 2n = 42, Charadriiformes) were the first set of chromosome-specific painting probes from Neoaves species that were able to cover the entire genome of a given bird species [24]. Thus, BOE painting probes have been used to establish genome-wide chromosomal homology among the karyotypes of eleven avian species in eight different orders [24–28]. The results demonstrate that BOE painting probes are robust tools for delineating avian chromosome evolution, especially for revealing the rearrangements that involve microchromosomes. Moreover, avian evolution are thought to involve three major events: (1) the divergence of Palaeognathae (ratites) and Neognathae (others), (2) the divergence from the Neognathae of the Galloanserae, and (3) the divergence of the remainder of Neognathae (Neoaves) [29, 30]. GGA belongs to the branch of the second divergence, which occurred about 88 million years, while BOE, ACI, EGA and NNI belong to Neoaves, their divergences completed about 50 million years ago [31]. Thus, BOE probes are better suited for comparative cytogenetic study in Neoaves than GGA probes due to the higher hybridization efficiency when applied to Neoaves species than GGA. In the present study, BOE painting probes were used to detect chromosomal homology in ACI, EGA and NNI, three ciconiiform species from two different families, and to define the origin of microchromosomes in the evolution of these species. To verify some of the hybridization results of BOE probes, three paint probes from GGA and the griffon vulture (*Gyps fulvus,* GFU) (GGA4, GFU 16 and GFU 14 + 27) were used in hybridizations with ACI and NNI chromosomes. Our study provides some new insights into the karyotype evolution and phylogenetic relationships in Ciconiiformes.

## Results

### Karyotype characteristics of ACI, EGA and NNI

**Fig. 1:**
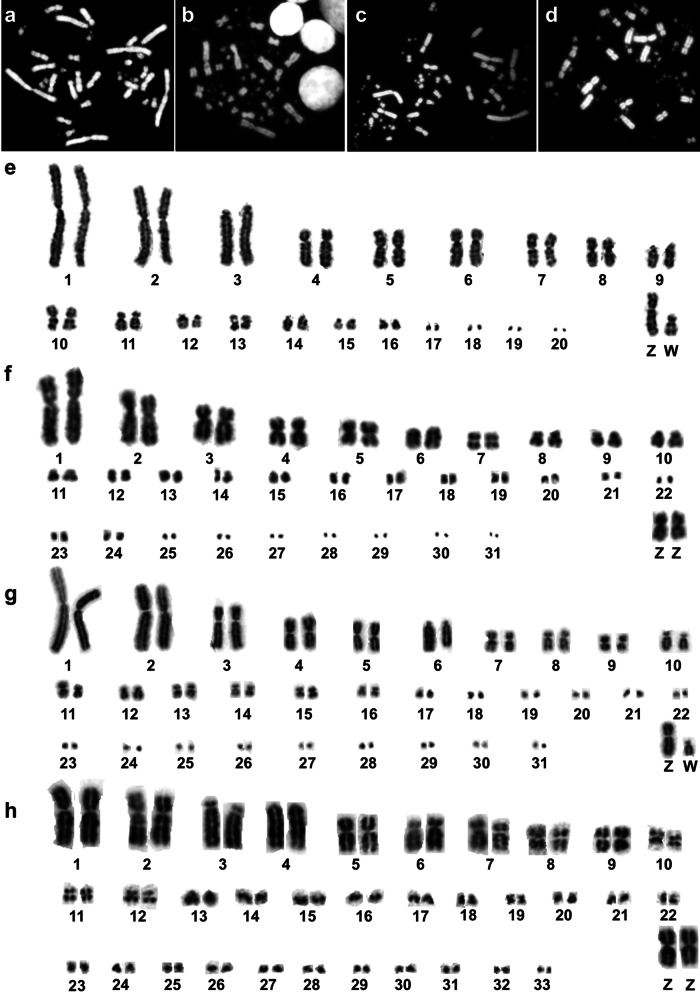
Metaphases and karyotypes of *Burhinus oedicnemus* (BOE, 2n = 42), *Ardea cinerea* (ACI, 2n = 64), *Egretta garzetta* (EGA, 2n = 64), and *Nipponia nippon* (NNI, 2n = 68) stained and banded with DAPI. **a** The metaphase of BOE. **b** The metaphase of ACI. **c** The metaphase of EGA. **d**. The metaphase of NNI. **e** The karyotype of BOE. **f** The karyotype of ACI. **g** The karyotype of EGA. **h** The karyotype of NNI

To facilitate comparisons, metaphases and karyotypes of avian species studied here are shown in Fig. 1, including BOE, the species from which the painting probes were derived (Fig. 1a and e). The karyotypes of ACI and EGA had been investigated previously [9, 10, 14, 17]. However, the chromosome numbers of these two species varied in different reports, with the diploid numbers ranging from 60 to 68, due to difficulties in determining the exact number of microchromosomes (MIC). Our chromosome counts support 64 as the modal diploid number of ACI (Fig. 1b) and EGA (Fig. 1c). The diploid number of NNI is 68 (Fig. 1d), as reported previously [12, 15, 32]. The karyotypes of ACI (Fig. 1f) and EGA (Fig. 1g) are similar, consisting of five pairs of large to medium-sized bi-armed chromosomes, one pair of medium-sized acrocentric chromosomes, ten pairs of medium to small-sized bi-armed chromosomes and fifteen pairs of dot-like microchromosomes. The karyotype of NNI (Fig. 1h) consists of three pairs of large bi-armed chromosomes, one pair of large telocentric chromosomes, eight pairs of medium-sized to small-sized bi-armed chromosomes, and twenty-one pairs of dot-like microchromosomes. The Z chromosome is a medium-sized metacentric in these three species (Fig. 1f–h), while the W chromosome is a small acrocentric in EGA (Fig. 1g). In comparison with karyotypes of GGA and BOE, the karyotypes of ciconiiform species have different number of medium to small-sized bi-armed chromosomes as found in BOE (Fig. 1 a and 1e), and the number of dot-like microchromosomes is lower than that of GGA.

### Hybridization of BOE probes onto chromosomes of ACI and EGA

**Fig. 2:**
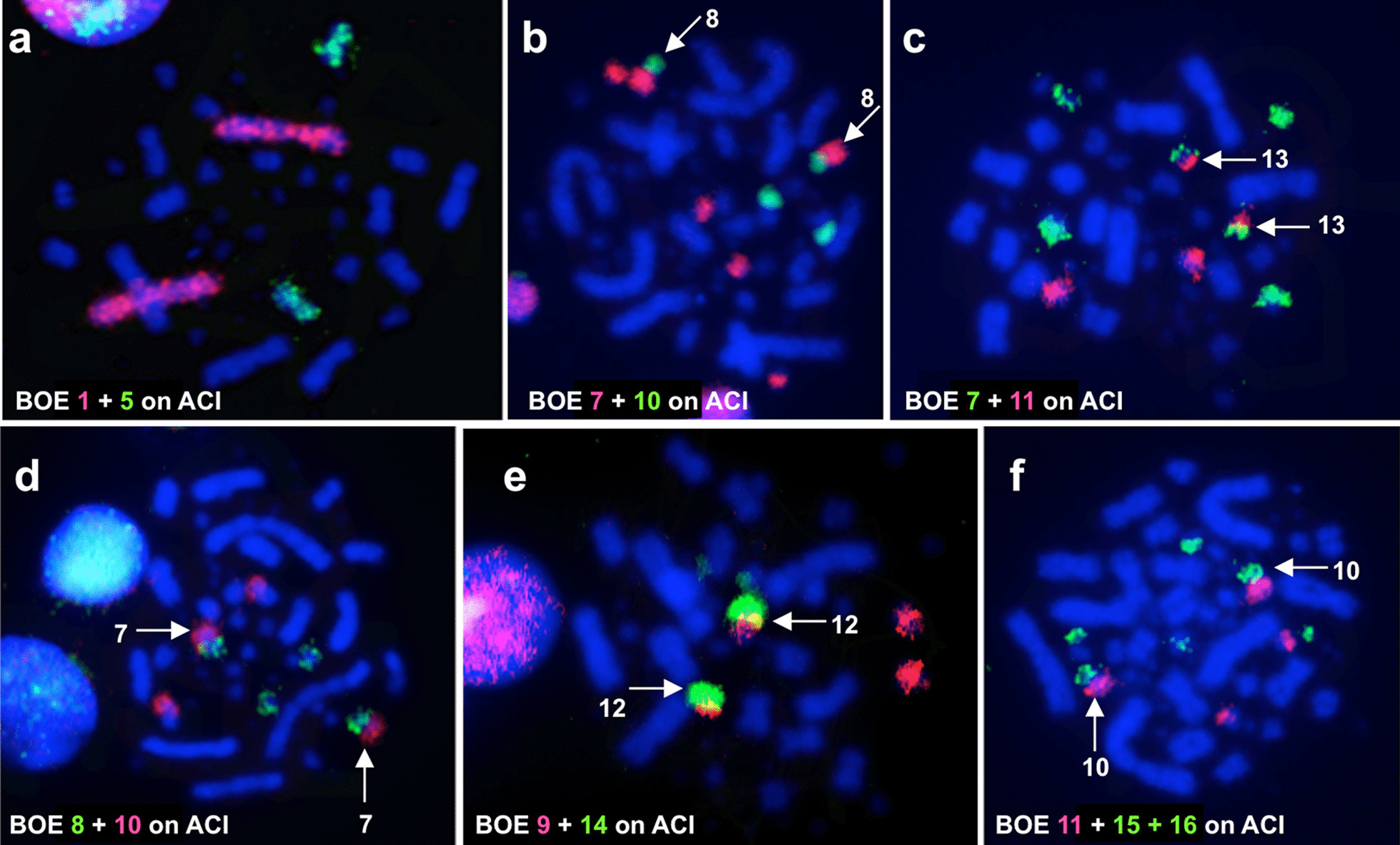
Dual-color FISH examples of ACI. **a** Hybridization of BOE 1 (red) and 5 (green) probes to ACI chromosomes. **b** Hybridization of BOE 7 (red) and 10 (green) probes to ACI chromosomes, arrows show ACI 8. **c** Hybridization of BOE 7 (green) and 11 (red) probes to ACI chromosomes, arrows show ACI 13. **d** Hybridization of BOE 8 (green) and 10 (red) probes to ACI chromosomes, arrows show ACI 7. **e** Hybridization of BOE 9 (red) and 14 (green) probes to ACI chromosomes, arrows show ACI 12. **f** Hybridization of BOE 11 (red) and 15 + 16 (green) probes to ACI chromosomes, arrows show ACI 10

**Fig. 3:**
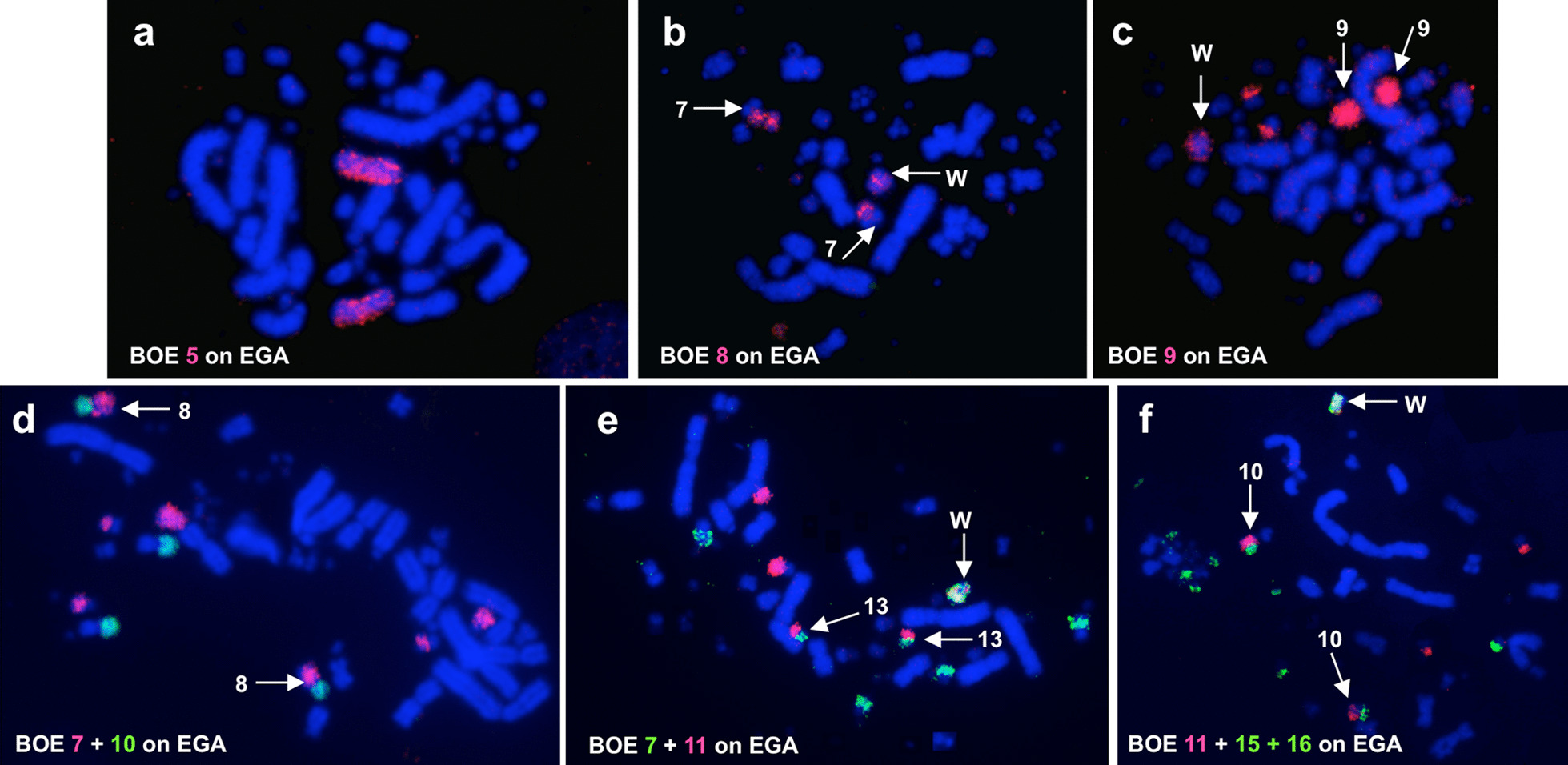
FISH examples of EGA. **a** Hybridization of BOE 5 (red) probe to EGA chromosomes. **b** Hybridization of BOE 8 (red) probe to EGA chromosomes, arrows show EGA 7. **c** Hybridization of BOE 9 (red) probe to EGA chromosomes, arrows show EGA 9. **d** Hybridization of BOE 7 (red) and 10 (green) probes to EGA chromosomes, arrows show EGA 8. **e** Hybridization of BOE 7 (green) and 11 (red) probes to EGA chromosomes, arrows show EGA 13. **f** Hybridization of BOE 11 (red) and 15 + 16 (green) probes to EGA chromosomes, arrows show EGA 10. Note: BOE probes 7, 9 and 15 + 16 also gave cross-hybridization signals to EGA W chromosome in b, c, e and f

**Fig. 4:**
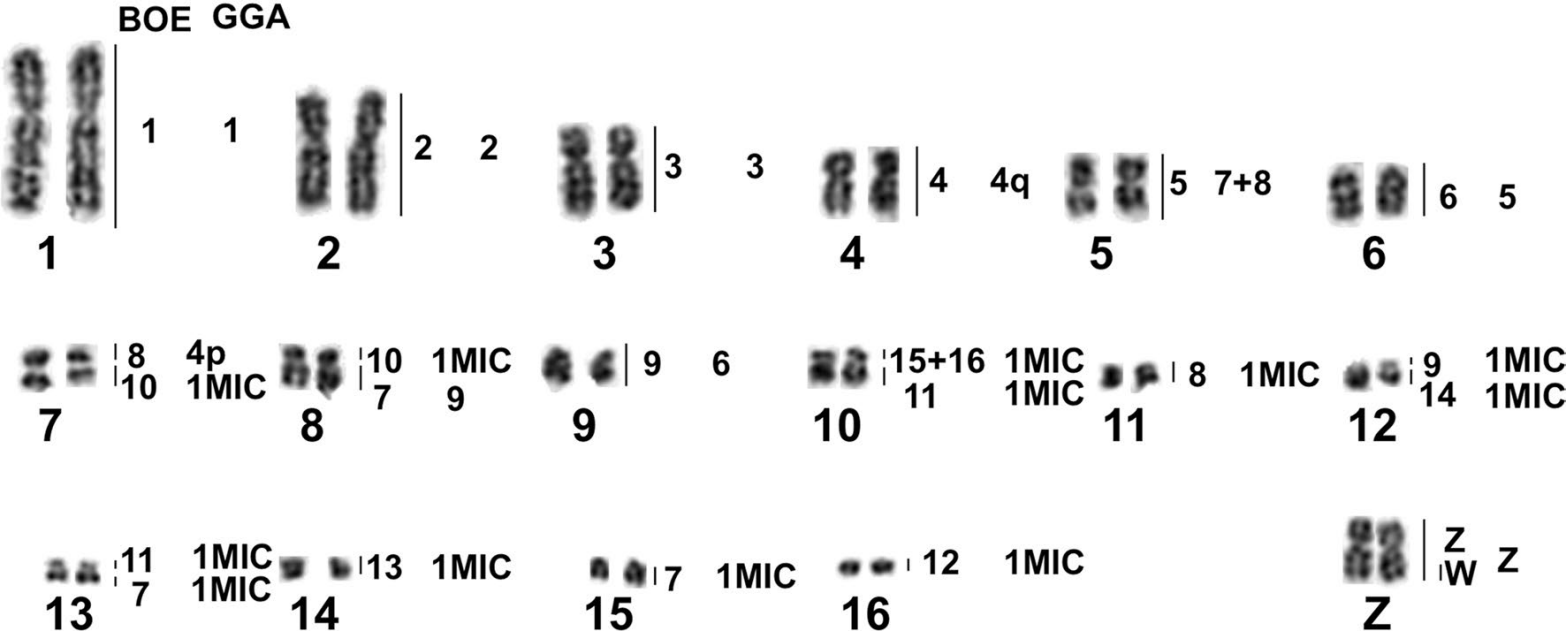
Partial DAPI-banded karyotype of ACI with assignment of homologies to BOE and GGA. p: the short arm of a chromosome; q: the long arm of a chromosome; MIC: microchromosome

**Fig. 5:**
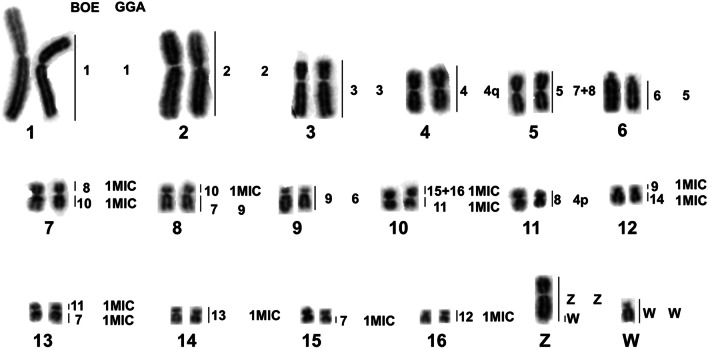
Partial DAPI-banded karyotype of EGA with assignment of homologies to BOE and GGA. p: the short arm of a chromosome; q: the long arm of a chromosome; MIC: microchromosome

The hybridization patterns produced by each BOE probe on metaphases of ACI and EGA are identical, confirming that ACI and EGA have similar karyotypes. Examples of BOE probes hybridizing onto chromosomes of ACI and EGA are shown in Figs. 2 and 3. The first six chromosome pairs of ACI and EGA are highly conserved, each showing homology with one BOE macrochromosome (BOE 1–6) (corresponding to GGA 1–3, 4q, 7 + 8 and 5) respectively (Figs. 2a and 3a). Five out of the ten smaller bi-armed chromosomes of ACI and EGA were each painted by two BOE probes and five syntenic homologous segment associations [BOE 7 + 10, 7 + 11, 8 + 10, 9 + 14 and 11 + (15 + 16)] (corresponding to GGA 9 + 1 MIC, 1MIC + 1MIC, 1 MIC + 1 MIC and 1 MIC + 1 MIC) are revealed in ACI (Fig. 2b–f) and EGA (Fig. 3b–f). The remaining five small bi-armed chromosomes of ACI and EGA each corresponds to one BOE segment (BOE 9q, 8p, 13q, 7q and 12q) (corresponding to GGA 6, 4p, and 3 MICs). Some dot-like microchromosomes in ACI and EGA are painted by one BOE probe, and some are not hybridized by any BOE probe. The Z chromosome probe of BOE hybridizes to the whole Z chromosomes of ACI and EGA, and the W chromosome probe of BOE paints the W chromosome and the distal part of the long arm of EGA Z (Zqter). The hybridization results of BOE probes on chromosomes of ACI and EGA are summarized in Figs. 4 and 5 and Table 1.

**Table 1:** Chromosomal correspondence between *Burhinus oedicnemus* (BOE), *Gallus gallus* (GGA)*, Ardea cinerea* (ACI), *Egretta garzetta* (EGA) and *Nipponia nippon* (NNI) revealed by chromosome painting with BOE chromosome-specific painting probes

BOE	GGA	ACI	EGA	NNI
1	1	1	1	2q, 4
2	2	2	2	1
3	3	3	3	3
4	4q	4	4	6
5	7, 8	5	5	5p + q(part)
6	5	6	6	7
7	9, 2 MICs	8q, 13q, 15q	8q, 13q, 15q	8q, 11, 1 MIC
8	4p, 1 MIC	7p, 11	7p, 11, W	9p, 10
9	6, 1 MIC	9, 12p	9, 12p, W	2p, 1 MIC
10	2 MICs	7q, 8p	7q, 8p, W	5 qter, 8p
11	2 MICs	10q, 13p	10q, 13p	9q, 1 MIC
12	2 MICs	16, 1 MIC	16, 1 MIC, W	12, 1 MIC
13	2 MICs	14, 1 MIC	14, 1 MIC, W	2 MICs, W
14	2 MICs	12q, 1 MIC	12q, 1 MIC	2 MICs
15, 16	3 MICs	10p, 2 MICs	10p, 2 MICs, W	3 MICs, Zpter
17, 18, 19, 20	1 MIC	~ 4 MICs	~ 4 MICs	~ 4 MICs
Z	Z	Z	Z, W	Z, W
W	W, Zqter	Zqter	W, Zqter	W, Zqter

*MIC* microchromosome; *p* the short arm of a chromosome; *q* the long arm of a chromosome; *pter* the distal of the short arm of a chromosome; *qter* the distal of the long arm of a chromosome

### Hybridization of BOE probes onto chromosomes of NNI

**Fig. 6:**
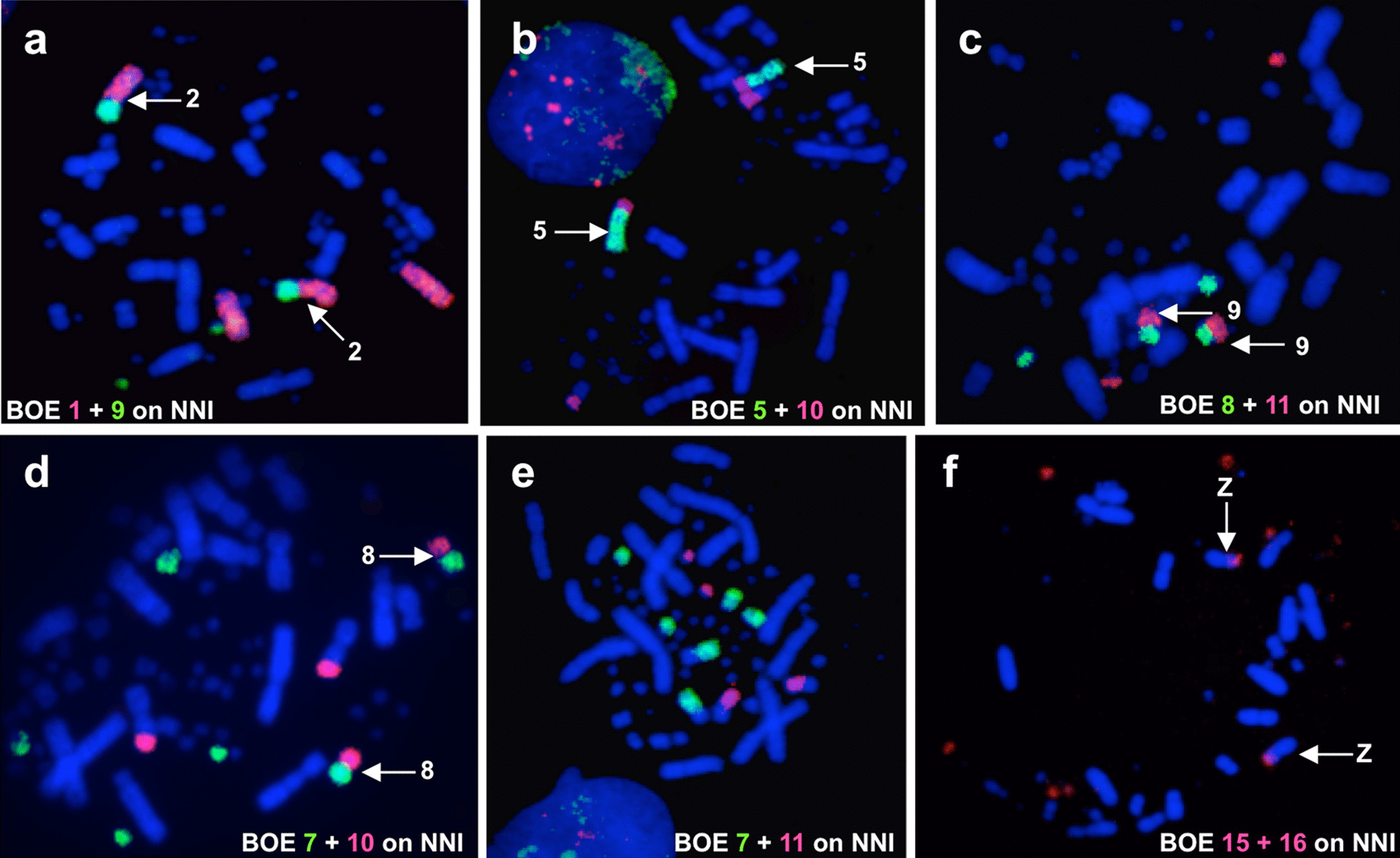
FISH examples of NNI. **a** Hybridization of BOE 1 (red) and 9 (green) probes to NNI chromosomes, arrows show NNI 2. **b** Hybridization of BOE 5 (green) and 10 (red) probes to NNI chromosomes, arrows show NNI 5. **c** Hybridization of BOE 8 (green) and 11 (red) probes to NNI chromosomes, arrows show NNI 9. **d** Hybridization of BOE 7 (green) and 10 (red) probes to NNI chromosomes, arrows show NNI 8. **e** Hybridization of BOE 7 (green) and 11 (red) probes to NNI chromosomes. **f** Hybridization of BOE 15 + 16 (red) to NNI chromosomes, arrows show NNI Z chromosomes with cross-hybridization signals

**Fig. 7:**
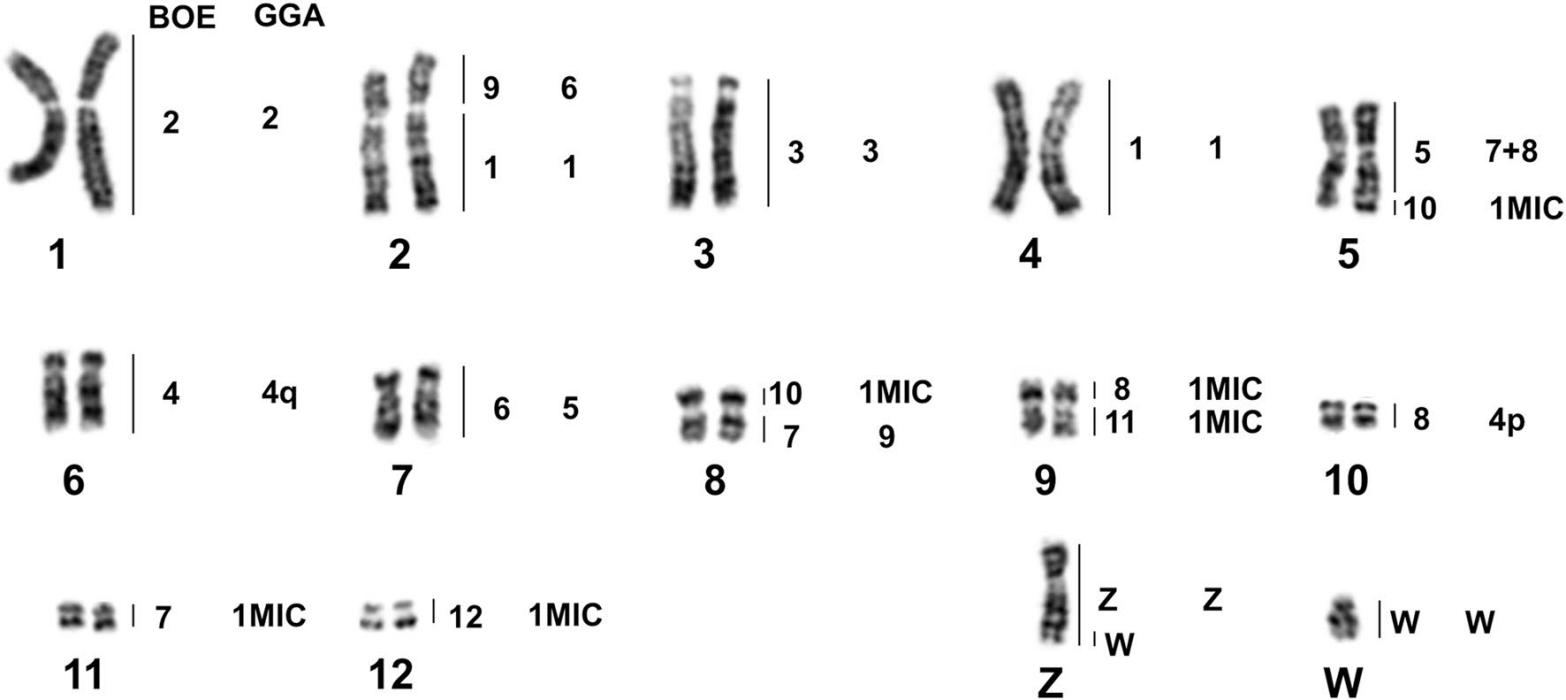
Partial DAPI-banded karyotype of NNI with assignment of homologies to BOE and GGA. p: the short arm of a chromosome; q: the long arm of a chromosome; MIC: microchromosome

Eighteen chromosome painting probes from BOE were hybridized to metaphases of NNI. All BOE probes produce distinctive signals on NNI chromosomes. FISH examples are shown in Fig. 6, and the results of chromosome painting between BOE and NNI are summarized in Fig. 7 and Table 1. The hybridization patterns of BOE probes in NNI (Fig. 7) are different from those observed in ACI (Fig. 4) and EGA (Fig. 5). A fission of BOE 1 (corresponding to GGA 1) homologue and a fusion of two BOE homologous segments (BOE 1p + 9q) (corresponding to GGA 1p + 6) are detected by BOE probes in NNI (Fig. 6a). Each of four NNI macrochromosomes is homologous to one BOE chromosome (BOE 2–4, 6) (corresponding to GGA 2, 3, 4q and 5). In addition, three syntenic associations of BOE segments (BOE 5 + 10, 7 + 10 and 8 + 11) [corresponding to GGA (7 + 8) + 1 MIC, 9 + 1 MIC and 1 MIC + 1 MIC] were found in the karyotype of NNI (Fig. 6b–d). The three smallest bi-armed chromosomes of NNI are each homologous to one BOE segment (BOE 8p, 7qpart and 13q) (corresponding to GGA 4p + 2 MICs). The pattern of hybridization results in NNI sex chromosomes and dot-like microchromosomes (Fig. 6f) are identical to the pattern shown in ACI and EGA mentioned above.

### Hybridization of three GGA and GFU probes onto chromosomes of ACI and NNI

**Fig. 8:**
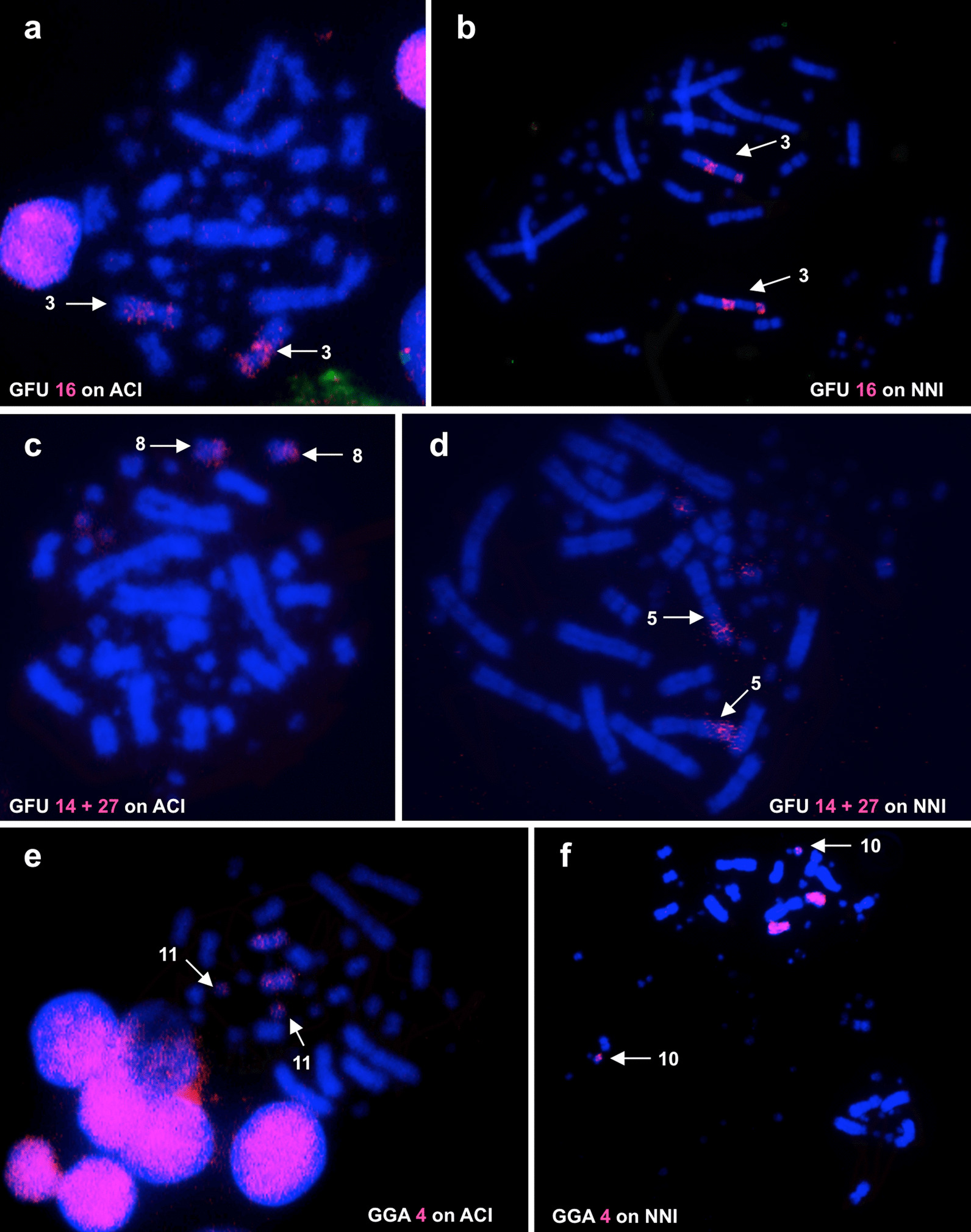
Chromosome painting using selected GGA and GFU probes to hybridize to chromosomes of ACI and NNI. **a** Hybridization of GFU 16 probe (red) to ACI chromosomes, arrows show ACI 3. **b** Hybridization of GFU 16 (red) probe to NNI chromosomes, arrows show NNI 3. **c** Hybridization of GFU 27 (red) probe (containing GFU 14) to ACI chromosomes, arrows show ACI 8. **d** Hybridization of GFU 27 (red) probe (containing GFU 14) to NNI chromosomes, arrows show NNI 5. **e** Hybridization of GGA 4 probe (red) to ACI chromosomes, arrows show ACI 11. **f** Hybridization of GGA 4 probe (red) to NNI chromosomes, arrows show NNI 10

To clarify previous cytogenetic results and to resolve some uncertainties about the interpretation of hybridization results of BOE probes on the origin of microchromosomes, we selected three GGA and GFU probes (GGA 4, GFU 16 and 14 + 27) to paint the chromosomes of ACI and NNI. Since ACI and EGA have similar karyotypes and identical hybridization patterns when painted with BOE probes, EGA is not included in this part of the study. Both ACI 3 (submetacentric) and NNI 3 (acrocentric) are painted by the probe of BOE 3 (corresponding to GGA 3) (see above). When we used the probe of GFU 16, which is homologous to part of BOE 3 [26], the distal part of the short arm of ACI 3 was not painted (Fig. 8a), while the short arm of NNI 3 was hybridized (Fig. 8b), confirming that a pericentric inversion had occurred in the ardeid ancestor. As mentioned above, BOE 8 and 10 probes each painted two segments of ACI and NNI chromosomes respectively, but we cannot determine the regional origin of each of these two homologous segments using the BOE whole chromosome paints. Previous studies indicated that GFU 27 was homologous to the short arm of BOE 10 (BOE 10p), and the short arm of GGA 4 (GGA 4p) corresponded to the short arm of BOE 8 (BOE 8p) [24, 26]. The hybridization of GFU 27 (sorted together with GFU 14) probe to chromosomes of ACI and NNI indicate that ACI 8p was homologous to BOE 10p (Fig. 8c), while the distal part of the short arm of NNI 5 was homologous to BOE 10p (Fig. 8d). As found in most avian species, the GGA 4 probe gave two signals on ACI and NNI chromosomes. GGA4p was homologous to one smaller chromosome of ACI (ACI 11, Fig. 8e) and NNI (NNI 10, Fig. 8f) respectively. These results clarify the above uncertainties using BOE probes and establish the chromosomal correspondence among BOE, GGA, ACI, EGA and NNI (Table 1 and Figs. 4, 5 and 7).

## Discussion

Previous studies have indicated that five pairs of BOE macrochromosomes (BOE 1–4, 6) are highly conserved. As found in many other avian species, the five largest ancestral avian chromosomes, homologous to GGA 1–3, 4q and 5, are each retained as a single intact BOE chromosome (BOE 1–4 and 6), while the other 10 pairs of BOE bi-armed chromosomes (BOE 5, 7–15) each represent a fusion product of two or three homologous GGA smaller macrochromosomes or microchromosomes [24]. Therefore, probes from medium-sized BOE chromosomes are ideal tools for studying evolutionary rearrangements involving microchromosomes in different avian species. Recently, bacterial artificial chromosome (BAC) probes from GGA and zebra finch (*Taeniopygia guttata*, Passeriformes) have been applied to investigate the chromosomal evolution of 41 species in 16 different orders [28, 33–41]. These studies indicate that most bird species so far studied have maintained an evolutionary stability in their microchromosome organization, except for species in a few orders (e.g., Falconiformes, Psittaciformes, Cuculiformes, and Suliformes), in which microchromosome fusions have been detected. In our study, microchromosome fusions were also observed in three ciconiiform species by using BOE probes, adding Ciconiiformes to this list of exceptions. Our results demonstrate that BOE probes and BAC mapping are alternative approaches for revealing avian microchromosome fusions. Although we failed to establish the complete microchromosome correspondences between BOE and the three ciconiiform species due to the difficulty in identifying avian dot-like microchromosomes, the BOE painting results in other avian species indicate the number of microchromosomes painted and the quantity of microchromosome fusions. We expect that more avian species will be studied by using BOE probes in the future.

Up to now, the karyotypes of five species in three ciconiiform families have been studied by chromosome painting using probes from GGA, LAL and BOE ([23], this study). Since the comparative chromosome map between BOE and GGA has been established [24], the chromosome correspondence among GGA, BOE and other avian species can be inferred. Despite the use of different probes in these studies, using GGA chromosome homology as the reference, the chromosomal rearrangements found in this study can be compared with those in other ciconiiform species and can shed light on their evolutionary relationships. Table 2 lists the homologous correspondence between GGA 1–9 and their counterparts in BOE and the five ciconiiform species so far studied. These data indicate that species from different ciconiiform families have experienced different chromosome rearrangements during evolution. For example, different numbers of chromosomal fusions (3, 5 and 6) have been detected in Ciconiidae, Ardeidae and Threskiornithidae.

**Table 2:** Homologies between GGA (*G. gallus*) 1–9 and chromosomes of BOE and five species in Ciconiiformes revealed by chromosome painting

Species	2n	Homologues of GGA 1–9									References
		1	2	3	4	5	6	7	8	9	
*Burhinus oedicnemus*, BOE	42	1	2	3	4,8p	6	9q	5q	5p	7p	[13,24]
*Ardea cinerea*, ACI	64	1	2	3	4, 11	6	9	5q	5p	8q	This study
*Egretta garzetta*, EGA	64	1	2	3	4, 11	6	9	5q	5p	8q	This study
*Nipponia nippon*, NNI	68	2q,4	1	3	6,10	7	2p	5q	5p	8q	This study
*Jabiru mycteria*, JMY	56	1	2	3	4,8q	5	6p	9	7q	7p	[23]
*Ciconia maguari*, CMA	72	1	2	3	4,8q	5	7q	9	6q	6p	[23]

*p* the short arm of a chromosome; *q* the long arm of a chromosome

Among the fusions detected in Ardeidae and Threskiornithidae, two (corresponding to GGA 7 + 8 and 9 + 1 MIC) seem to be common for species in our study. BOE 5 (corresponding to GGA 7 + 8) as a conserved synteny was detected in ACI (Fig. 2a), EGA (Fig. 3b) and NNI. Furthermore, the BOE 5 homologous segment was fused to one homologous segment from part of BOE 10 (corresponding to one GGA MIC) to form NNI 5 (Fig. 6b). Besides these three ciconiiform species, to date this association of GGA 7 + 8 is found only in BOE and the Southern lapwing (*Vanellus chilensis*), two species belonging to Charadrii which is one of the three major clades of Charadriiformes [24, 42]. Including BOE and *V. chilensis*, five species in Charadriiformes have been studied by chromosome painting; this association is not detected in the other three species of Charadriiformes [25, 27, 43]. Therefore, the GGA 7 + 8 fusion could have happened independently in Charadrii and the three ciconiiform species since these lineages (Charadriiformes and Ciconiiformes) are distantly related according to phylogenetic studies [30, 31, 44]. This fusion could be a common cytogenetic character linking Ardeidae with Threskiornithidae. The fusion of BOE 7 + 10 (corresponding to GGA 9 + 1 MIC) was found in ACI (Fig. 2b), EGA (Fig. 3d) and NNI (Fig. 6d), but our painting results with the probe of GFU 27 (corresponding to BOE 10p) indicate that this is not a true common character for Ardeidae and Threskiornithidae, because the microchromosome fused to the GGA 9 homologue is different in these three species (Fig. 8c and d).

In Ciconiidae, three fusions (corresponding to GGA 8 + 9, 6 + 1 MIC and 4p + 1 MIC) were observed in two Brazilian stork species, and one of the fusions (corresponding to GGA 8 + 9) was considered to be a shared character for storks [23]. In our study, the fusions of GGA 8 + 9 and 6 + 1 MIC were not detected in the three ciconiiform species, while the painting results using the GGA 4 probe indicated that GGA 4p was homologous to one whole microchromosome in ACI (Fig. 8e) and NNI (Fig. 8f) and not included in a fusion. No shared fusions have been found yet in the species of Ciconiidae and other ciconiiform families (Ardeidae and Threskiornithidae).

Although only five ciconiiform species have been studied by chromosome painting to date, these results demonstrate that storks differ from herons and ibises in chromosome rearrangements, and herons and ibises share a syntenic association that indicates a closer karyotype relationship. These chromosome painting data also provide support for the molecular phylogenomic studies that place herons and ibises in one clade and storks in another clade [30, 44–49]. To further elaborate the karyotype relationships among different ciconiiform families, chromosome painting data are needed from more ciconiiform species and from Pelecaniformes, an order which shows some karyotypic similarities and a closer phylogenetic relationship with Ciconiiformes.

## Conclusions

We have established genome-wide comparative chromosome maps between BOE and three ciconiiform species from two different families by chromosome painting using a set of BOE probes that cover the entire BOE genome. Our results indicate that chromosomal fusions play an important role in the karyotype evolution of species in Ciconiiformes. Besides fusions between macro- and microchromosome, fusions between microchromosomes are also found in species from Ardeidae and Threskiornithidae, leading to some small bi-armed chromosomes. A fusion of segments homologous to GGA 7 and 8 is shared by these three ciconiiform species, indicating that this fusion may be a cytogenetic signature linking Ardeidae and Threskiornithidae, and reinforcing the proposition that places herons and ibises in one clade as reported in previous molecular phylogenomic studies.

## Methods

### Cell culture, metaphase preparation and chromosome nomenclature

Three species belonging to two different families in Ciconiiformes, the eastern grey heron (*A. cinerea*, ACI), the little egret (*E. garzetta*, EGA) and the crested ibis (*N. nippon*, NNI) were studied. The fibroblast cell lines of EGA (KCB 2011136, **♀** and 2012017, **♂**) and NNI (KCB 2011119, ♂ and 2012055S, ♀) were provided by Kunming Cell Bank, the Chinese Academy of Sciences. The cells were grown in DMEM (Dulbecco's Modified Eagle Medium) enriched with 15% fetal bovine serum. Metaphase preparation and slide preparation of NNI and EGA were carried out following conventional methods. Mitotic chromosomes of ACI were obtained from the bone marrow suspension of a male ACI prepared and stored in 1999 in Kunming Cell Bank, the Chinese Academy of Sciences.

The karyotype of ACI and EGA were arranged according to the relative length of the chromosomes from the largest to the smallest. The chromosomes of NNI were numbered according to previously published karyotypes with a minor revision [12, 15, 32].

### FISH, image capture and processing

Painting probes from the stone curlew (*B. oedicnemus*, BOE) were hybridized onto metaphases of ACI, EGA and NNI respectively to delineate the homologous segments between BOE, ACI, EGA and NNI. In addition, three painting probes from the chicken (*G. gallus,* GGA) and the griffon vulture (*G. fulvus,* GFU) were used to verify results from BOE probes. The preparation of BOE, GGA and GFU painting probes, cross-species chromosome painting, image capture and processing were carried out as previously described [24, 26]. In brief, the whole set of BOE painting probes and part of GFU and GGA painting probes were generated from flow-sorted chromosomes and amplified by degenerate oligonucleotide-primed polymerase chain reaction (DOP-PCR). Painting probes was labeled with either Biotin-16-dUTP or digoxigenin-11-dUTP (Roche, Basel, Switzerland) in a second round of PCR amplification and visualized using Cy3-avidin (Amersham), mouse anti-digoxigenin monoclonal antibody (Sigma, D8156) and goat anti-mouse FITC conjugate antibody (Sigma, F0257). Hybridization signals (homologous to BOE chromosomes or chromosomal segments) were assigned to specific chromosomes or chromosome regions of ACI, EGA and NNI defined by inverted DAPI-banding patterns. After single color FISH, dual-color FISH was performed to further verify associations between different conserved segments in the rearranged chromosomes of ACI, EGA and NNI. To facilitate comparison of homologous chromosomal segments across bird species, the homologous GGA chromosome segments as inferred from the GGA–BOE comparative chromosomal map [24] were indicated beside ACI, EGA and NNI chromosomes.

## Data Availability

All data supporting the results reported in this article can be found at the article itself. No additional dataset is available.

## Abbreviations

GGA: *Gallus gallus*
LAL: *Leucopternis albicollis*
ACI: *Ardea cinerea*
EGA: *Egretta garzetta*
NNI: *Nipponia nippon*
BOE: *Burhinus* *o**edicnemus*
2n: Diploid number
GFU: *Gyps fulvus*
MIC: Microchromosome
DAPI: 4′, 6-Diamidino-2-phenylindole
BAC: Bacterial artificial chromosome
JMY: *Jabiru mycteria*
CMA: *Ciconia maguari*
KCB: Kunming cell bank
DMEM: Dulbecco's Modified Eagle Medium
FISH: Fluorescence in situ hybridization
p: The short arm of a chromosome
q: The long arm of a chromosome
